# Intravitreal bevacizumab for choroidal neovascular membrane associated with Best's vitelliform dystrophy

**DOI:** 10.4103/0301-4738.60096

**Published:** 2010

**Authors:** Ekta Rishi, Pukhraj Rishi, Sheshadri Mahajan

**Affiliations:** Shri Bhagwan Mahavir Vitreoretinal Services, Sankara Nethralaya, 18, College Road, Chennai - 600 006, India

**Keywords:** Best's disease, bevacizumab, choroidal neovascular membrane, vascular endothelial growth factor, vitelliform macular dystrophy

## Abstract

Best's vitelliform macular dystrophy is a hereditary form of progressive macular dystrophy that can be complicated by choroidal neovascularization. Authors report successful treatment of choroidal neovascularization with intravitreal bevacizumab in one such eye in an ‘adult’ Indian male with visual improvement. A 23-year-old male presented with diminution of vision in the right eye for the past sixteen months. Visual acuity was 20/400 in the that eye. After three consecutive intravitreal injections of bevacizumab (1.25 mg/0.05 ml), vision improved to 20/120. Seven months following the last injection of bevacizumab, fundus appeared stable and visual acuity was maintained. No drug-related ocular or systemic side effects were encountered. To the best of our knowledge (PubMed search), this is the first report of its kind in an adult Indian patient. Intravitreal bevacizumab appears to be a promising and cost-effective modality of treatment in such eyes with potential for improvement in vision. However, a long-term follow-up is warranted.

Best's disease, also known as Best's vitelliform macular dystrophy, is a hereditary form of progressive macular dystrophy that can be complicated by choroidal neovascularization.[1] Andrade *et al*. have reported regression of choroidal neovascularization, resolution of exudative manifestations and significant visual improvement following a single treatment session of photodynamic therapy (PDT).[2] However, PDT may not always be an affordable treatment option in the developing world. Bevacizumab, an anti-vascular endothelial growth factor (VEGF), is a humanized, monoclonal antibody being commonly used as an ‘off-label’ drug for the management of choroidal neovascularization due to age related macular degeneration,[3] pathological myopia,[4] idiopathic parafoveal telengiectasia[5] and angioid streaks.[6] Recently, there has been a report of treatment of choroidal neovascularization related to Best's disease in a thirteen-year-old child with a single injection of intravitreal bevacizumab.[7] We hereby report a case of successful treatment of choroidal neovascularization with intravitreal bevacizumab in one such eye in a young adult Indian male associated with visual improvement. To the best of our knowledge (PubMed search), this is the first report of its kind.

## Case Report

**Figure 1:**
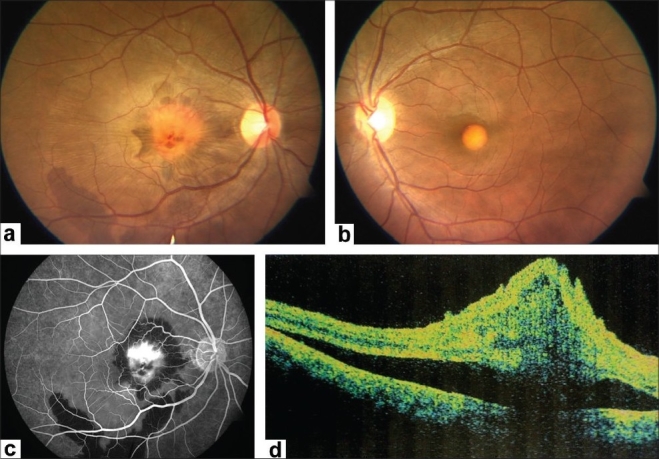
(At presentation) (a) Fundus examination of the right eye shows a subfoveal vitelliform lesion associated with a yellowish-grey choroidal neovascular membrane, sub-retinal and sub-retinal pigment epithelium hemorrhages extending up to the inferotemporal vascular arcade. Subretinal fluid and internal limiting membrane striae are also noted. (b) Left eye shows an egg yolk appearance at the fovea characteristic of Best's disease. (c) Right eye fundus fluoresceine angiography reveals intense leakage from the choroidal neovascular membrane. Blocked fluorescence corresponds to the subretinal and sub-RPE hemorrhages. (d) Optical coherence tomography of right eye shows presence of SRF, cystoid macular edema, fresh subretinal hemorrhage and disorganized RPE-choriocapillaris complex suggestive of choroidal neovascular membrane

A 23-year-old male presented with a history of diminution and distortion of vision in the right eye for the past 16 months. His best corrected visual acuity (BCVA) was 20/400; < N36 in right eye and 20/20; N6 in the left. Biomicroscopic examination of the anterior segment was unremarkable in both eyes. Fundus examination of the right eye revealed a large, hypopigmented, egg yolk-like subfoveal lesion with fresh subretinal hemorrhage, subretinal fluid (SRF) and internal limiting membrane (ILM) striae [Fig. 1a]. These clinical findings were suggestive of choroidal neovascular membrane (CNVM) in the right eye. Left eye fundus examination also revealed a hypopigmented, egg yolk-like lesion at the fovea, characteristic of the vitelliform stage of Best's disease [Fig. 1b]. Fundus fluorescein angiography (FFA) revealed early hyperfluorescence with intense late leakage confirmatory of CNVM in the right eye along with hypofluorescence corresponding to the subretinal hemorrhage [Fig. 1c]. Electro-oculogram (EOG) of both eyes revealed sub-normal responses; however recordings were unreliable due to lack of sustained fixation during the test. Optical coherence tomography (OCT) of the right eye confirmed the presence of SRF, cystoid macular edema, disorganization of the retinal pigment epithelium (RPE)-choriocapillaris complex corresponding to the CNVM, vitelliform changes and subretinal blood [Fig. 1d]. The treatment options of PDT and anti-VEGF agents were discussed with the patient. The patient opted for intravitreal bevacizumab over PDT and the same was administered (1.25 mg/0.05 ml) under strict aseptic precautions, after obtaining an informed (written) consent. Post injection period was uneventful.

**Figure 2:**
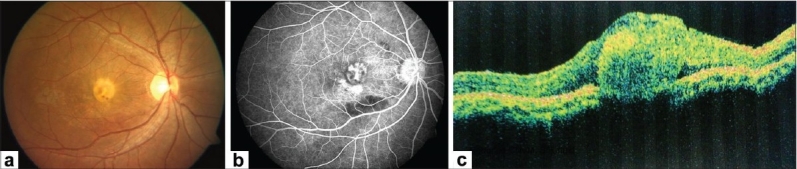
(5 weeks after first injection of bevacizumab) (a) Right eye fundus shows regressing choroidal neovascular membrane with marked resolution of subretinal hemorrhage and reduction of subretinal fluid at fovea. (b) Fundus fluorescein angiography shows reduced leakage from choroidal neovascular membrane in right eye. (c) Optical coherence tomography shows involuting choroidal neovascular membrane and significant reduction in subretinal fluid

**Figure 3:**
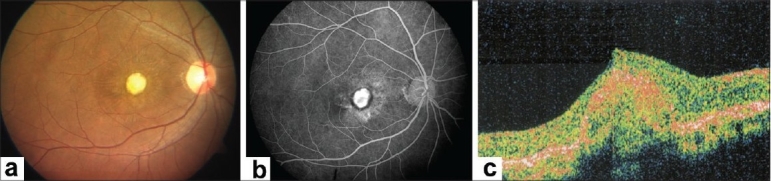
(8 weeks after third injection of bevacizumab) (a). Fundus examination of right eye showed a scarred choroidal neovascular membrane with internal limiting membrane striae. (b). Fundus fluorescein angiography shows absence of leakage and mere staining of the choroidal neovascular membrane in right eye. (c). Optical coherence tomography shows fibrosed choroidal neovascular membrane with complete resolution of subretinal fluid

**Figure 4:**
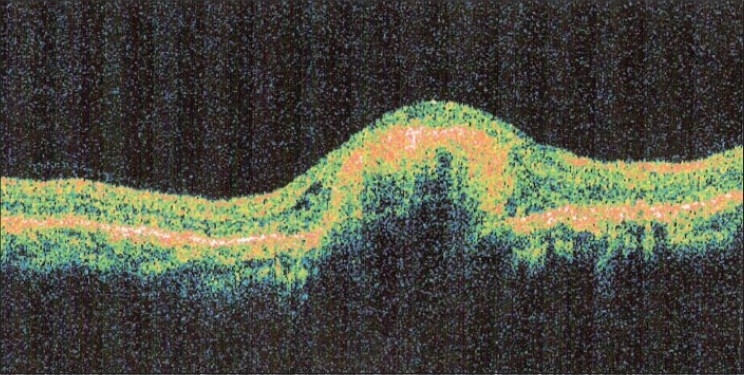
(12 weeks after third injection of bevacizumab) Optical coherence tomography confirms a completely regressed choroidal neovascular membrane with no recurrence of subretinal fluid

At five weeks follow up, BCVA in the right eye was still 20/400, N36 and the left eye was stable. Fundus examination of the right eye revealed regression of CNVM with marked resolution of subretinal hemorrhage and reduction of SRF at fovea [Fig. 2a]. FFA revealed reduced leakage from CNVM [Fig. 2b]. OCT revealed markedly reduced SRF and increased fibrosis of CNVM as compared to the previous visit [Fig. 2c]. A second intravitreal injection of bevacizumab (1.25 mg/0.05 ml) was given at six weeks following the first one. Post injection period was uneventful. Four weeks later, BCVA in the right eye was still maintained. Fundus examination revealed a scarred CNVM with ILM folds, resolution of subretinal fluid and subretinal hemorrhage. FFA showed reduced yet persisting leakage from the CNVM in the right eye. Clinical findings were confirmed on OCT. Hence, a third injection of intravitreal bevacizumab was given. Post injection period was uneventful. The patient reported at eight weeks following the third injection. Right eye BCVA had improved to 20/120, N12. Anterior segment examination was normal in both eyes. Fundus examination of the right eye showed a scarred CNVM with ILM striae [Fig. 3a]. There was no evidence of SRF and subretinal hemorrhage had completely resolved. Clinical findings were confirmed on FFA [Fig. 3b] and OCT [Fig. 3c]. The eye remained stable at 12 weeks follow-up [Fig. 4] and subsequently, at the last follow-up, seven months after the third injection of bevacizumab. During the entire course of treatment, we did not encounter any drug-related ocular or systemic side effects.

## Discussion

Our report corroborates the results of similar treatment as reported by Leu *et al*.[7] in a 13-year-old boy, *albeit* in an adult Indian patient with three injections. We also feel that had the treatment been initiated at a more acute stage, the outcome would have been better. Intravitreal bevacizumab appears to be a promising, cost-effective modality of treatment with a potential for improvement in visual acuity, although with inherent risks associated with intravitreal injections. A long-term follow-up is warranted to address possible recurrences and determine the optimal number of re-treatments required in achieving a long-term stabilization of the aforesaid condition.

## References

[1] Miller SA, Bresnick GH, Chandra SR (1976). Choroidal neovascular membrane in Best's vitelliform macular dystrophy. Am J Ophthalmol.

[2] Andrade RE, Farah ME, Costa RA (2003). Photodynamic therapy with verteporfin for subfoveal choroidal neovascularization in Best's disease. Am J Ophthalmol.

[3] Yoganathan P, Deramo VA, Lai JC, Tibrewala RK, Fastenberg DM (2006). Visual improvement following intravitreal bevacizumab (Avastin) in exudative age-related macular degeneration. Retina.

[4] Tewari A, Dhalla MS, Apte RS (2006). Intravitreal bevacizumab for treatment of choroidal neovascularization in pathologic myopia. Retina.

[5] Jorge R, Costa RA, Calucci D, Scott IU (2007). Intravitreal bevacizumab (Avastin) associated with the regression of subretinal neovascularization in idiopathic juxtafoveolar retinal telangiectasis. Graefes Arch Clin Exp Ophthalmol.

[6] Teixeira A, Moraes N, Farah ME, Bonomo PP (2006). Choroidal neovascularization treated with intravitreal injection of bevacizumab (Avastin) in angioid streaks. Acta Ophthalmol Scand.

[7] Leu J, Schrage NF, Degenring RF (2007). Choroidal Neovascularisation secondary to Best's disease in a 13-year-old boy treated by intravitreal bevacizumab. Graefe's Arch Clin Exp Ophthalmol.

